# Successful implementation of ChIP-seq antibody quality control at Diagenode using automated ChIP protocol on the SX-8G IP-Star^®^ Compact

**DOI:** 10.1186/1756-8935-6-S1-P133

**Published:** 2013-04-08

**Authors:** Ignacio Mazon, Sharon Squazzo, Jan Hendrickx, Géraldine Goens, Catherine D’andrea, Geoffrey Berguet, Celine Sabatel, Miklos Laczik, Dominique Poncelet

**Affiliations:** 1 Diagenode S.A., CHU, Tour GIGA B34, 3ème étage 1 Avenue de I’Hôpital, 4000 Liège, Sart-Tilman, Belgium; 2 Diagenode Inc., 400 Morris Avenue, Suite 101, Denville, NJ 07834, USA

## 

Chromatin immunoprecipitation (ChIP) is the most widely used method to study protein-DNA interactions. A successful ChIP, however, is largely depending on the use of well characterized, highly specific ChIP-grade antibodies.

ChIP-seq has become the gold standard for whole-genome mapping of protein-DNA interactions. The generalized adoption of this technology is currently limited by four main technical hurdles. First, the reproducibility and biological relevance of DNA-associated protein landscapes depend on the specificity and performance of the antibodies in the context for which they are used. Second, the ChIP-seq method requires optimized protocols ensuring high recovery and increased signal-to-noise ratio. Third, as an effort to reduce the cost per sample and improve reproducibility, the ChIP-seq method should be compatible with automation. Finally, the economical and widespread use of ChIP-seq requires access to a fast and high value/quality next-generation sequencing platform. Here, we demonstrate the successful use of the Diagenode integrated line of products to establish a QC procedure to qualify antibodies and standardize ChIP-seq experiments.

